# Numerical modeling on hybrid nanofluid (Fe_3_O_4_+MWCNT/H_2_O) migration considering MHD effect over a porous cylinder

**DOI:** 10.1371/journal.pone.0251744

**Published:** 2021-07-01

**Authors:** Zahir Shah, Anwar Saeed, Imran Khan, Mahmoud M. Selim, Ikramullah, Poom Kumam

**Affiliations:** 1 Department of Mathematical Sciences, University of Lakki Marwat, Lakki Marwat, Khyber Pakhtunkhwa, Pakistan; 2 Center of Excellence in Theoretical and Computational Science (TaCS-CoE), Faculty of Science, King Mongkut’s University of Technology Thonburi (KMUTT), Thung Khru, Bangkok, Thailand; 3 Department of Mathematics, Bacha Khan University, Charsada, Khyber Pakhtunkhwa, Pakistan; 4 Department of Mathematics, Al-Aflaj College of Science and Humanities Studies, Prince Sattam bin Abdulaziz University, Al-Kharj, Al-Aflaj, Saudi Arabia; 5 Department of Mathematics, Suez Faculty of Science, Suez University, Suez, Egypt; 6 Department of Physics, Kohat University of Science & Technology, Kohat, Khyber Pakhtunkhwa, Pakistan; 7 Fixed Point Research Laboratory, Fixed Point Theory and Applications Research Group, Center of Excellence in Theoretical and Computational Science (TaCS-CoE), Faculty of Science, King Mongkut’s University of Technology Thonburi (KMUTT), Thung Khru, Bangkok, Thailand; 8 Department of Medical Research, China Medical University Hospital, China Medical University, Taichung, Taiwan; Central University of Karnataka, INDIA

## Abstract

The free convective hybrid nanofluid (Fe_3_O_4_+MWCNT/H_2_O) magnetized non-Darcy flow over a porous cylinder is examined by considering the effects constant heat source and uniform ambient magnetic field. The developed coupled PDEs (partial differential equations) are numerically solved using the innovative computational technique of control volume finite element method (CVFEM). The impact of increasing strength of medium porousness and Lorentz forces on the hybrid nanofluid flow are presented through contour plots. The variation of the average Nusselt number (Nu_ave_) with the growing medium porosity, buoyancy forces, radiation parameter, and the magnetic field strength is presented through 3-D plots. It is concluded that the enhancing medium porosity, buoyancy forces and radiation parameter augmented the free convective thermal energy flow. The rising magnetic field rises the temperature of the inner wall more drastically at a smaller Darcy number. An analytical expression for Nusselt number (Nu_ave_) is obtained which shows its functional dependence on the pertinent physical parameters. The augmenting Lorentz forces due to the higher estimations of Hartmann retard the hybrid nanoliquid flow and hence enhance the conduction.

## 1. Introduction

The enhancement of heat energy transmission rate is an attractive research topic due to its central importance in heat energy technology like heat reservoirs, electronic cooling, solar collectors, nuclear reactors cooling, and heat exchangers and so on. There are different modes of heat energy transportation, which consist of convection, radiation and conduction. In convection, the heat energy transfer takes place due to the collective motion of the heated fluid within different parts of the system. The convection is considered as forced or free. The free convection happens due to the buoyancy-driven forces which emerge because of the distinction in temperature gradients and densities inside the liquid. The convective heat energy transfer has been remained an attractive research field because of its wide scope of uses in engineering and industry, Sowmya et al. [1], Nehad Ali Shah et al. [2], Kassai et al. [3]. The application of an external magnetic field changes the liquid flow shape. The Lorentz force due emerges the presence of the field lessens the liquid speed, subsequently diminishes the convection. The liquid dynamics are mostly governed by the magnitude and trend of the practical fields. The MHD flow has various uses in different industrial processes, like in nuclear energy reactors, crystal growth, electronic and electrical devices, solar energy technology, magnetic confinement fusion, and so on. The various parts of the MHD convection flow are explored both theoretically and experimentally by different analysts Farhad et al. [4], Haq et al. [5], Sohail et al. [6]. The main disadvantage of an ordinary fluid is its low thermal energy carrying capability. To overcome this, researchers have introduced in the recent past innovative fluids, termed nanofluids. The Nanofluids are generally obtained by mixing nanoparticles of metals (metal oxides) with the base liquids. The dimensions and physical sketch of the nanoparticles play a central character in enhancing the thermal conductivities of regular liquids. The use of nanoliquid augments the effective thermal conduction and hence the thermal energy transfer coefficient. The idea of the nanomaterial addition with the classical fluid to augment its thermal conductivity was introduced for the first time by Choi and Eastman [7]. Pak and Cho [8] studied experimentally the impacts of γ-alumina (Al_2_O_3_) and titanium dioxide on the turbulent heat energy transportation of water. These authors found that the mixing of nanoparticles with water fallouts in the enhancement of the convective heat energy transformation coefficient. Rashidi and Nezamabad [9] experimentally examined the carbon nanotubes nanofluids thermal energy transfer coefficient by considering the impact of constant heat flux. They gained a prominent development in its heat transfer coefficient and found its functional dependence on the axial distance. The conductivities of graphene and graphene oxides nanofluids are investigated by Mahanta and Abramson [10] and establish that the advanced thermal conductivity of multilayered graphene is because of the covalent interlayers bonding due to the presence of oxygen atoms. Sun et al. [11] explored the heat transmission characteristics of Ferro-nanofluids flowing over copper tubes and acquired that the blending of dispersants improved altogether the nanofluid stability. The trial examinations of the temperature transmission growth through the carbon nanotubes nanoliquids turbulent flow is done by Walvekar et al. [12]. Li et al. [13] explored a continuous thin layer of unsteady MHD liquid stream and transfer of nanofluid heat in the company of heat production (Generation) and thermophoresis factors. Mabood et al. [14] studied the boundary layer flow of two-dimensional MHD hybrid nanofluid through a flat stretching surface with thermal radiation influences. The stability examination with dual result utilizing the shooting technique for fluid flow with slip effects was investigated by Dero et al. [15]. During their study, they showed that the perpendicular magnetic field depreciates the stream function by a large amount as compared to the inclined and horizontal magnetic fields. More significant investigations regarding the nanoliquid flow can be obtained in the refs [16–19]. Nehad Ali Shah *et al* [20] studies the dynamics of hybrid nanofluids by use of type I and type II hybrid models with main importance on the modification.

Currently, there is advancement in looking at the heat energy change characteristics of hybrid nanofluids, which are gotten by mixing more than one sort of nanoparticles in the ordinary liquid. Hybrid nanofluids show an assortment of physicals, chemical and thermal characteristics that don’t have by a single segment nanofluid. Various dynamics of hybrid nanofluids have been explored by Taylor et al. [21], Hamza and Hafiz [22], and Said et al. [23]. The tremendous amount of experimental, analytical, and numerical research work affirms that hybrid nanoliquid is more reasonable and significant when contrasted with the simple one. The hybrid nanofluids are the advanced fluids that have uses in the heat energy transfer procedure, for example in microfluidizers, transportation, defense, fabrication etc. The copper-alumina nanoparticles by using the two-step method were analyzed by Suresh et al. [24]. Momin [25] investigated analytically the various aspects of diverse convective laminar hybrid nanoliquid flow in a tending cylinder. Sundar et al. [26] inspected the greater temperature energy transfers and the friction feature of the hybrid nano liquid. Suresh et al. [27] similarly examined and discussed the benefits of hybrid nano powder for the thermal energy system. Hayat and Nadeem [28] studied the thermal energy transfer properties of Ag-CuO/water nanoliquid. They obtained that, hybrid nanoliquid shows a superior heat transfer rate in contrast with the ordinary nanofluid. Usman et al. [29] examined the hybrid nanoliquid (Cu-Al_2_O_3_/water) movement over a porous field by seeing the effects of variable thermal conductivity and nonlinear radiation by using LSM. Mohebbi et al. [30] played out a two-dimensional (2D) mathematical simulation to examine the influence of MWCNT +Fe_3_O_4_/water nanoliquid on the thermal presentation of the vaulted passage with separated segments of cooling and heating. Safaei et al. [31] applied Artificial Neural Network and Shooting Technique to examine the effects produced due to the varying concentration and temperature on the thermal conduction of ZnO-TiO_2_/EG hybrid nanofluid. Lunde et al. [32] used Tiwari and Das model to perform the stability analysis and to discover the different solutions during the hybrid nanoliquid flow through a dwindling surface. Ghalambaz et al. [33] discussed the different aspects during the melting of nanoparticles heightened phase change materials by considering the effects of hybrid nanoparticles. Minna et al. [34] has set a review in which he portrays the improvement of the hybrid nanofluid and their advantages. Sandeep et al. [35] addressed strengthened thermal performance incorporated with inorganic nanomaterials in liquid film stream of non-Newtonian nanoliquids. The thermal efficiency of nanocomposites is supposed to be determined by influences including the base fluid’s heat capacity and thermal ability, the flowrate, the nanofluid’s solubility, the amount of colloidal matter as well as their proportions, and the flow structure. Very recently, Babazadeh et al. [36] analyzed the impacts of Lorentz forces and radiation on the hybrid nanofluid flow over a permeable inclusion by utilizing the CVFEM. Scientists tried to find new ways for improving the efficiency of system, Yanget et al. [37], Wang et al. [38], Hu et al. [39], Sheikholeslami et al. [40].

Ashwinkumar [41] analyzed the MHD movement of water based aluminum alloy with thermal diffusion and thermal radiations effects. Tlili et al. [42] studied the MHD flow of hybrid nanoliquid through a stretching surface with non-uniform thickness and slip impacts. Sheikholeslami et al. [43] investigated the effect of thermo-phoresis and Brownian moment on the Magnetohydrodynamic nanofluid identified by using FEM. Mabood et al. [44] scrutinized the MHD stagnation point flow of hybrid nanofluid flow with variable properties. Sulochana and Ashwinkumar [45] presented the convected flow of nanofluid past an extending surface considering the influences of Brownian movement and thermophoresis effects.

Liu et al. [46] presented the comparative study of 29 nm CuO and 47 nm Al2O3 containing motile microorganisms through a horizontal surface. Mahanthesh et al. [47] investigated the incompressible nanofluid flow through a rotating disk with Brownian movement and thermophoresis effect. Animasaun et al. [48] presented the significance of chemical reaction in an electrically conducting dusty fluid flow. Animasaun et al. [49] examined the buoyance effect on water based 47nm alumina nanofluid with non-linear thermal radiation. Reducing the price of final system is main goal of design in each system, Sheikholeslami et al [50].

After gaining the impetus from the above research studies, we want to analyze the non-Darcy hybrid nanofluid motion over a spongy cylinder by seeing the impacts of the heat source and ambient magnetic field. The flow is modeled through coupled PDEs which are numerically solved by employing CVFEM. In segment 2, the problem description is described. Section 3 develops the problem formulation and describes the simulation technique. The mesh analysis and code validation are discussed in section 4. The numerical results are obtained and enlightened over contour plots and 3-D diagrams in segment 5. The main research outcomes are concluded in the last section.

## 2. Research methodology

**Fig 1:**
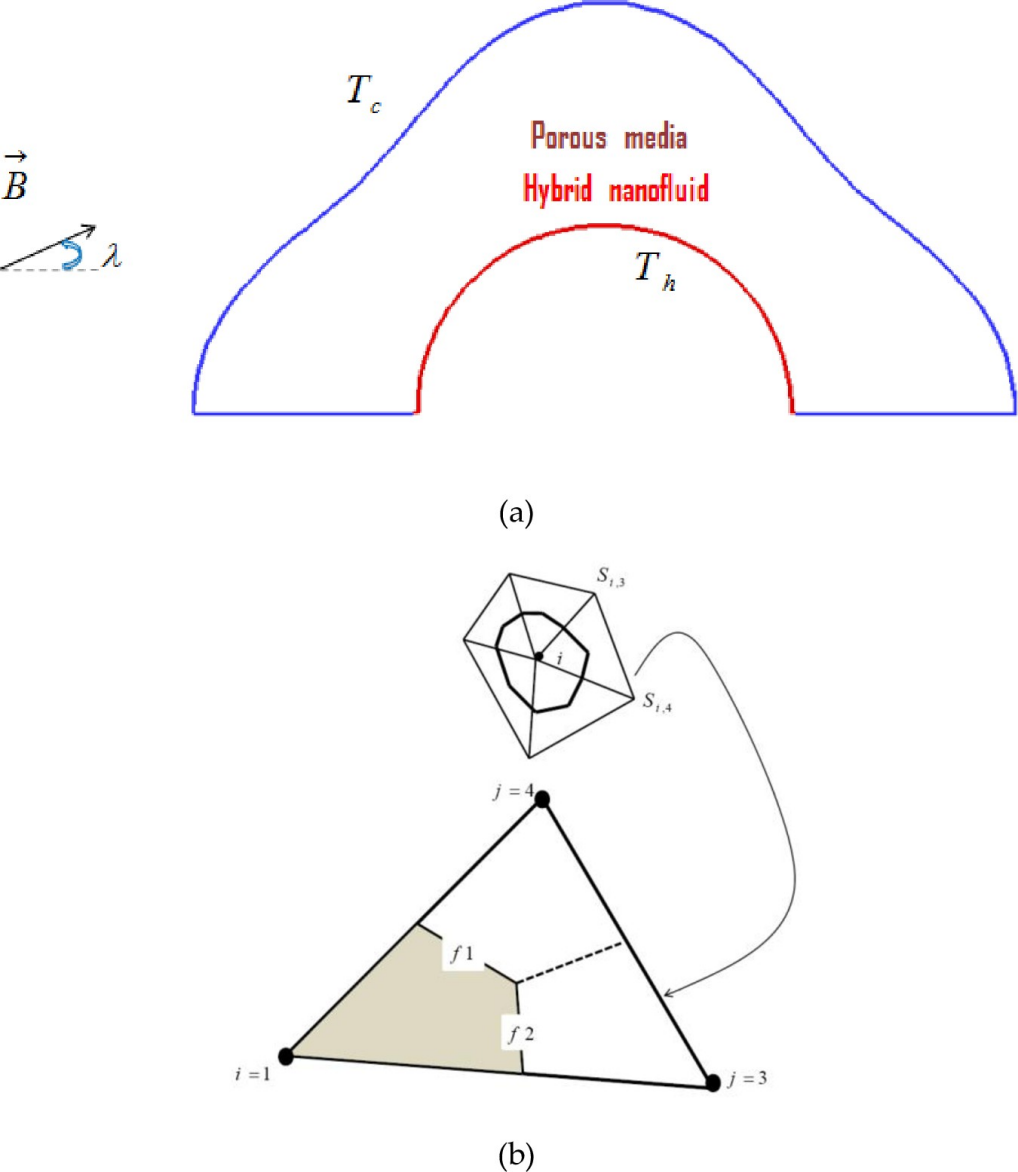
(a) Permeable container filled with hybrid nanofluid (b) CVFEM element.

Fig 1A and 1B displays the physical configuration of the present investigations and the triangular element for the simulation. It consists of a permeable cylinder filled with the hybrid nanofluid. The interior surface is sustained hot because of constant heat flux and the outer one is kept cold. The flow is controlled through an ambient uniform magnetic field. The governing formulation and the simulation technique for the non-Darcy hybrid nanofluid flow in the occurrence of the external uniform magnetic field and a constant heat source are briefly explained in this section.

## 3. Mathematical formulation of the governing equation

In the current investigation, we consider laminar 2-D (two dimensional) free convection non-Darcy MHD nanofluid flow under the effect of a constant magnetic field and heat source through a porous cylinder. The non-Darcy model is working for the permeable medium. The 2-D flow is modeled by the following coupled PDEs:

$$\frac{\partial v}{\partial y}+\frac{\partial u}{\partial x}=0$$ (1)

$$\begin{array}{l} u\frac{\partial u}{\partial x}+v\frac{\partial u}{\partial y}=\frac{\mu_{nf}}{\rho_{nf}}(\frac{\partial^{2}u}{\partial x^{2}}+\frac{\partial^{2}u}{\partial y^{2}})-\frac{1}{\rho_{nf}}\frac{\partial P}{\partial x}-\frac{1}{\rho_{nf}}\frac{\mu_{nf}}{K}u-(T_{c}-T)\beta_{nf}g\sin\gamma \\ +\sigma_{nf}B_{0}^{2}\left[-u{\left(\sin\lambda \right)}^{2}+v(\sin\lambda )(\cos\lambda )\right] \end{array}$$ (2)

$$\begin{array}{l} u\frac{\partial v}{\partial x}+v\frac{\partial v}{\partial y}=\frac{\mu_{nf}}{\rho_{nf}}(\frac{\partial^{2}v}{\partial x^{2}}+\frac{\partial^{2}v}{\partial y^{2}})-(T_{c}-T)\beta_{nf}g\cos\gamma -\frac{\partial P}{\partial y}\frac{1}{\rho_{nf}}-\frac{1}{\rho_{nf}}\frac{\mu_{nf}}{K}v\\ +\sigma_{nf}B_{0}^{2}\left[-v{\left(\cos\lambda \right)}^{2}+u(\sin\lambda )(\cos\lambda )\right] \end{array}$$ (3)

$$\begin{array}{l} \frac{1}{{\left(\rho C_{p}\right)}_{nf}}\frac{\partial q_{r}}{\partial y}+(u\frac{\partial T}{\partial x}+v\frac{\partial T}{\partial y})=k_{nf}(\rho C_{p}{)}_{nf}^{-1}(\frac{\partial^{2}T}{\partial y^{2}}+\frac{\partial^{2}T}{\partial x^{2}}),\\ \left[q_{r}=-\frac{4\sigma_{e}}{3\beta_{R}}\frac{\partial T^{4}}{\partial y},T^{4}≅4T_{c}^{3}T-3T_{c}^{4}\right]. \end{array}$$ (4)

Where, T, P, *ρ*, and *C*_*p*_ denote the temperature, pressure, density and heat capacity of the fluid, respectively. The symbols B_x_, B_y,_ u and v are respectively the magnetic field and fluid velocity x and y components.

The literature shows that there does not exist general relation which can be used to compute the thermo-physical characteristics of hybrid nanofluid. Therefore, we use in this study the experimental data about the hybrid nanofluid (MWCNT + Fe_3_O_4_ + H_2_O) characteristics from ref. [41] and are displayed in Table 1. The characteristics of MWCNT nanoparticles and Fe_3_O_4_ are described in Table 2 [42]. The relations for computing various nanofluid characteristics are:

**Table 1:** Characteristics of hybrid MWCNT–Fe_3_O_4_ / water nanofluid [41].

*ϕ*	μ(*mPa*.*s*)	*C*_*p*_(*j*/*kgk*)	*k*(*W*/*m*.*k*)	*ρ*(*kg*/*m*^*3*^)
0.003	1.01	4183.99	0.6856	1010.04
0	0.79	4182	0.602	998.5

**Table 2:** Characteristics of hybrid (MWCNT and Fe_3_O_4_) nanomaterials [42].

	*β*×*10*^*5*^(*K*^−*1*^)	*C*_*p*_(*j*/*kgk*)	*σ*(*Ω*⋅*m*)^−*1*^	*k*(*W*/*m*.*k*)	*ρ*(*kg*/*m*^*3*^)
Fe_3_O_4_	1.3	670	25000	6	5810
MWCNT	4.2	711	10^−7^	3000	2100


$$\begin{array}{l} \rho_{nf}\beta_{nf}=(1-\varphi )(\rho \beta {)}_{bf}+\varphi (\rho \beta {)}_{np},\beta_{np}=\frac{\beta_{MWCNT}\varphi_{MWCNT}+\beta_{Fe_{3}O_{4}}\varphi_{Fe_{3}O_{4}}}{\varphi_{MWCNT}+\varphi_{Fe_{3}O_{4}}}\\ \sigma_{nf}=\sigma_{bf}\left(1+\frac{3(\frac{\sigma_{np}}{\sigma_{bf}}-1)\varphi }{(\frac{\sigma_{np}}{\sigma_{bf}}+2)-(\frac{\sigma_{np}}{\sigma_{bf}}-1)\varphi }\right) \end{array}$$ (5)

From ref. [50], $\sigma_{np}=\sigma_{Fe_{3}O_{4}}$.

We consider the following formulation (Eq 6) in order to eliminate the pressure terms:

$$\begin{array}{l} \omega +\frac{\partial u}{\partial y}-\frac{\partial v}{\partial x}=0,\frac{\partial \psi }{\partial x}=-v,\frac{\partial \psi }{\partial y}=u \end{array}$$ (6)

We use the following relations to transform the model equations to non-dimensional form:

$$\begin{array}{l} U=\frac{uL}{\alpha_{nf}},V=\frac{vL}{\alpha_{nf}},\theta =\frac{T-T_{c}}{\Delta T},\Delta T=\frac{q^{\prime \prime }L}{k_{f}},(X,Y)=\frac{\left(x,y\right)}{L}\\ ,\Psi =\frac{\psi }{\alpha_{nf}},\Omega =\frac{\omega L^{2}}{\alpha_{nf}} \end{array}$$ (7)

Accordingly, Eqs (1–4) become:

$$\frac{\partial^{2}\Psi }{\partial Y^{2}}+\frac{\partial^{2}\Psi }{\partial X^{2}}=-\Omega ,$$ (8)

$$\begin{array}{l} U\frac{\partial \Omega }{\partial X}+\frac{\partial \Omega }{\partial Y}V=\mathrm{Pr}\frac{A_{5}}{A_{1}}\frac{A_{2}}{A_{4}}\left(\frac{\partial^{2}\Omega }{\partial Y^{2}}+\frac{\partial^{2}\Omega }{\partial X^{2}}\right)\\ +\mathrm{Pr}Ha^{2}\frac{A_{6}}{A_{1}}\frac{A_{2}}{A_{4}}\left(\frac{\partial U}{\partial X}\cos\lambda \sin\lambda -\frac{\partial V}{\partial X}{\left(\cos\lambda \right)}^{2}+\frac{\partial U}{\partial Y}{\left(\sin\lambda \right)}^{2}-\frac{\partial V}{\partial Y}\cos\lambda \sin\lambda \right)\\ +\mathrm{Pr}Ra\frac{A_{3}A_{2}^{2}}{A_{1}A_{4}^{2}}\left(\frac{\partial \theta }{\partial X}\cos\gamma -\frac{\partial \theta }{\partial Y}\sin\gamma \right)-\frac{\mathrm{Pr}}{Da}\frac{A_{5}}{A_{1}}\frac{A_{2}}{A_{4}}\Omega , \end{array}$$ (9)

$$(1+\frac{4}{3}{\left(\frac{k_{nf}}{k_{f}}\right)}^{-1}Rd)\frac{\partial^{2}\theta }{\partial Y^{2}}+(\frac{\partial^{2}\theta }{\partial X^{2}})=-\frac{\partial \theta }{\partial Y}\frac{\partial \Psi }{\partial X}+\frac{\partial \Psi }{\partial Y}\frac{\partial \theta }{\partial X}$$ (10)

The following definitions are employed in Eqs (9) and (10):

$$\begin{array}{l} \mathrm{Pr}=\upsilon_{f}/\alpha_{f},Ra=g(\rho \beta {)}_{f}\Delta TL^{3}/(\mu_{f}\alpha_{f}),Ha=LB_{0}\sqrt{\sigma_{f}/\mu_{f}}\\ A_{1}=\frac{\rho_{nf}}{\rho_{f}},A_{2}=\frac{{\left(\rho C_{p}\right)}_{nf}}{{\left(\rho C_{p}\right)}_{f}},A_{3}=\frac{{\left(\rho \beta \right)}_{nf}}{{\left(\rho \beta \right)}_{f}},\\ A_{4}=\frac{k_{nf}}{k_{f}},A_{5}=\frac{\mu_{nf}}{\mu_{f}},A_{6}=\frac{\sigma_{nf}}{\sigma_{f}} \end{array}$$ (11)

To solve the set of Eqs (8–10), the imposed boundary restrictions are stated as:

$$\begin{array}{l} \theta =1.0\;on\;left\;wall\\ \theta =0.0\;on\;right\;wall\\ \frac{\partial \theta }{\partial n}=0.0\;top\;and\;bottom\;walls\\ \Psi =0.0\;on\;all\;walls \end{array}$$ (12)

To examine the heat energy transformation rate, the relations used for computing Nu_loc_ (local Nusselet number) and Nu_ave_ (average Nusselet number) are:

$$Nu_{loc}=\frac{\partial \theta }{\partial n}(1+\frac{4}{3}{\left(\frac{k_{nf}}{k_{f}}\right)}^{-1}Rd)(\frac{k_{nf}}{k_{f}})$$ ((13))

$$Nu_{ave}=\frac{1}{S}\int_{0}^{s}Nu_{loc}ds$$ ((14))

### 3.1. Numerical simulation

Sheikholeslami [50] developed the simulation technique of CVFEM for solving heat energy transfer problems during the fluid flow. This method combines the FEM and FVM (finite element and volume methods). A trilateral component with lined interpolation is used for calculating the scalars as shown in Fig 1B. In the final step of scalars computation, the Gauss-Seidel technique is utilized.

## 4. Mesh analysis and results validation

**Fig 2:**
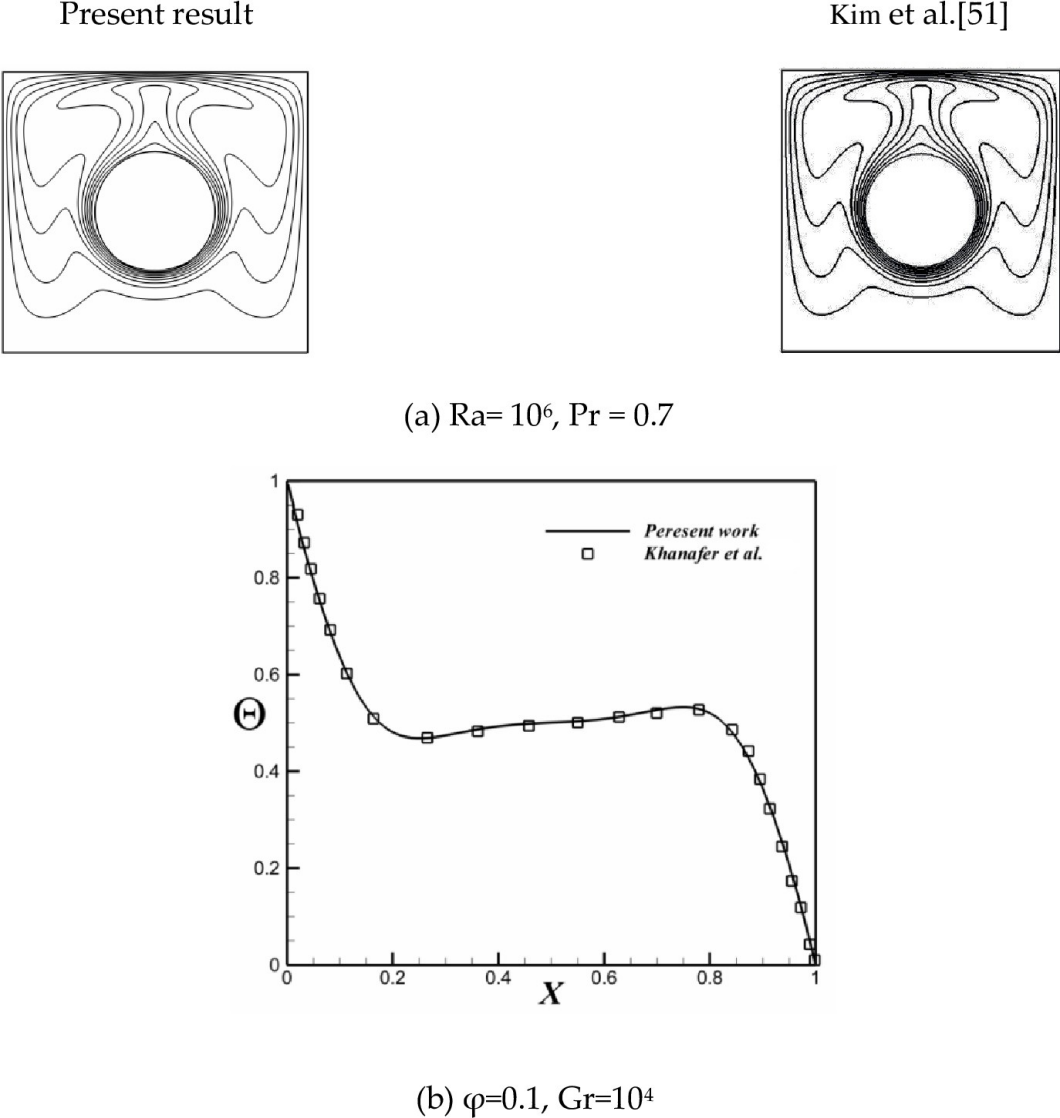
Validation for (a) free convection (Kim et al. [51]) (b) nanofluid (Khanafer et al. [52]).

The reliable outputs shall not depend on the mesh size. Therefore, the mesh analysis is carried out to reach the stage where the outputs are independent of the mesh dimensions. Table 3 shows the results of different mesh dimensions. To examine the accuracy of the present simulation code (CVFEM), it is used to simulate the previous studies [40, 50]. Fig 2 and Table 4 demonstrate the accuracy of the employed computational technique.

**Table 3:** Difference of *Nu*_*ave*_ with alteration of mesh size at *Ra* = 10^5^, *Rd* = 0.8,*Da* = 100,*Ha* = 20 and *ϕ* = 0.003.

*Mesh size in radial direction×angular direction*				
*51×151*	*61×181*	*71×211*	*81×241*	*91×271*
9.6983	9.7005	9.7026	9.7035	9.7071

**Table 4:** Variation of *Nu*_*ave*_ with varying Ha at Pr = 0.733.

*Ha*	*Gr* = *2*×*10*^*5*^	
	Present	Rudraiah et al. [53]
50	2.67911	2.8442
10	4.9047	4.8053

## 5. Results and discussion

**Fig 3:**
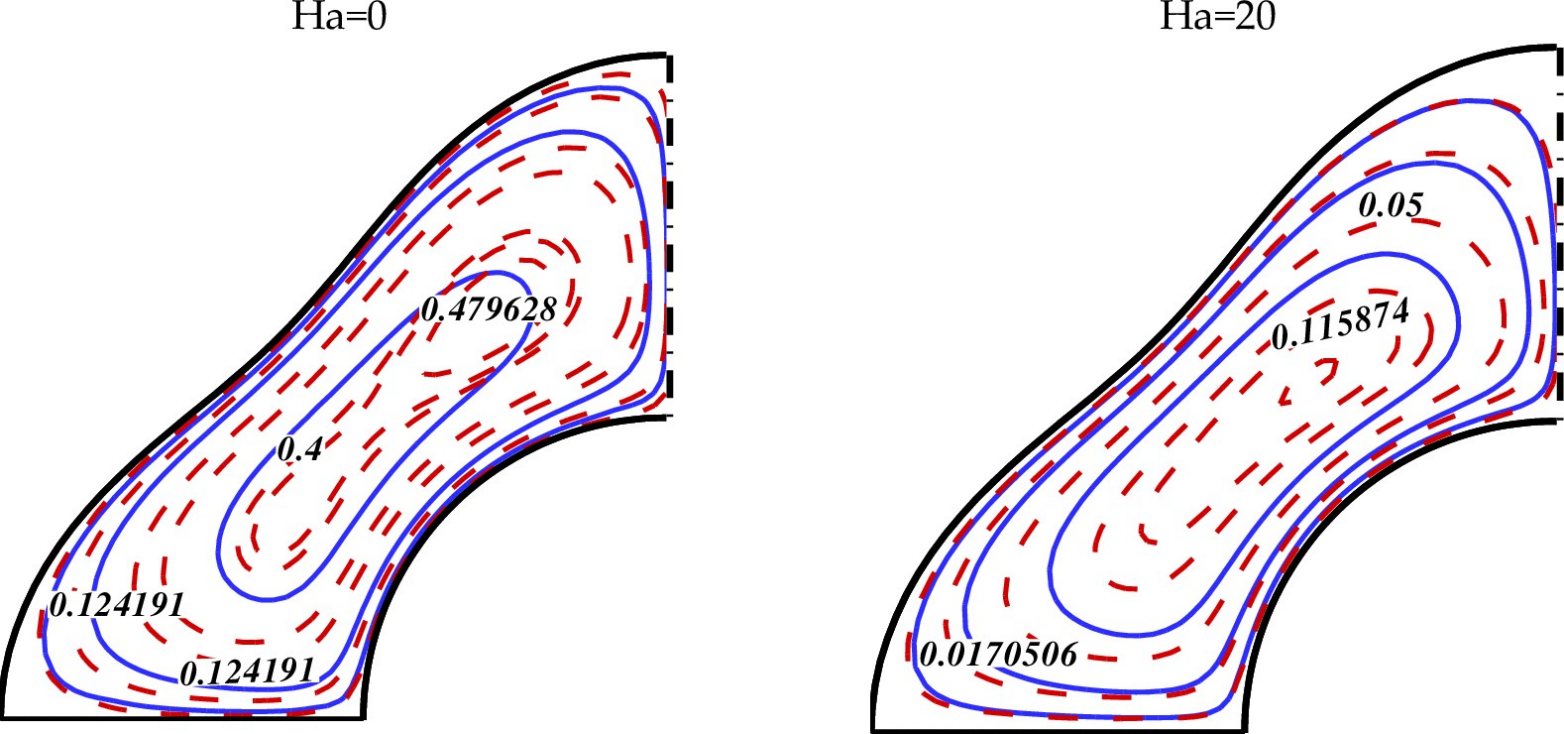
Effect of *Da* on nanofluid behavior (*Da* = *100* (––) and *Da* = *0*.*01* (-—-)) when *Ra* = *10*^*3*^,*Rd* = *0*.*8*.

**Fig 4:**
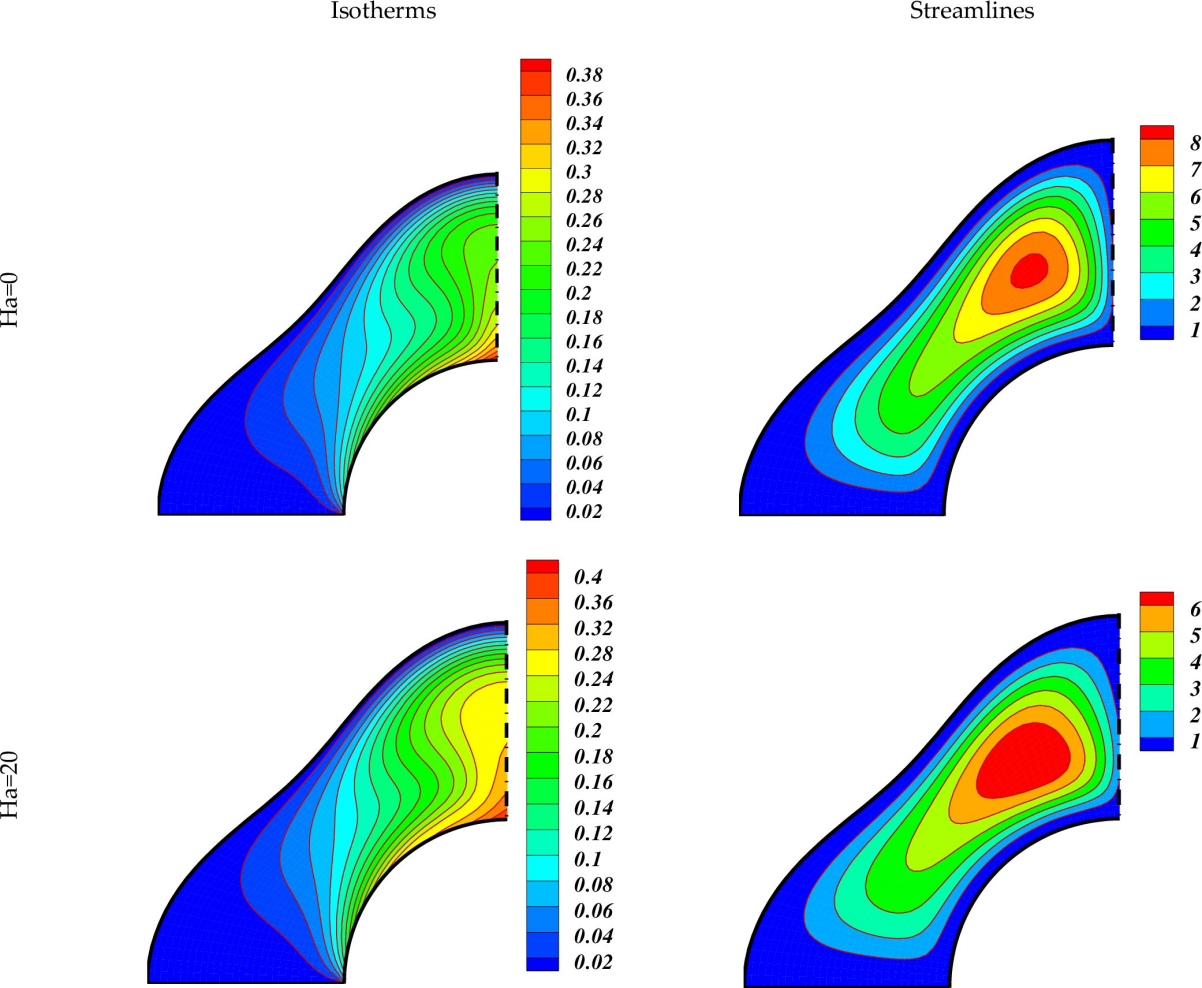
Impact of Ha on the flow when *Ra* = *10*^*5*^,*Rd* = *0*.*8*,*Da* = *0*.*01*.

**Fig 5:**
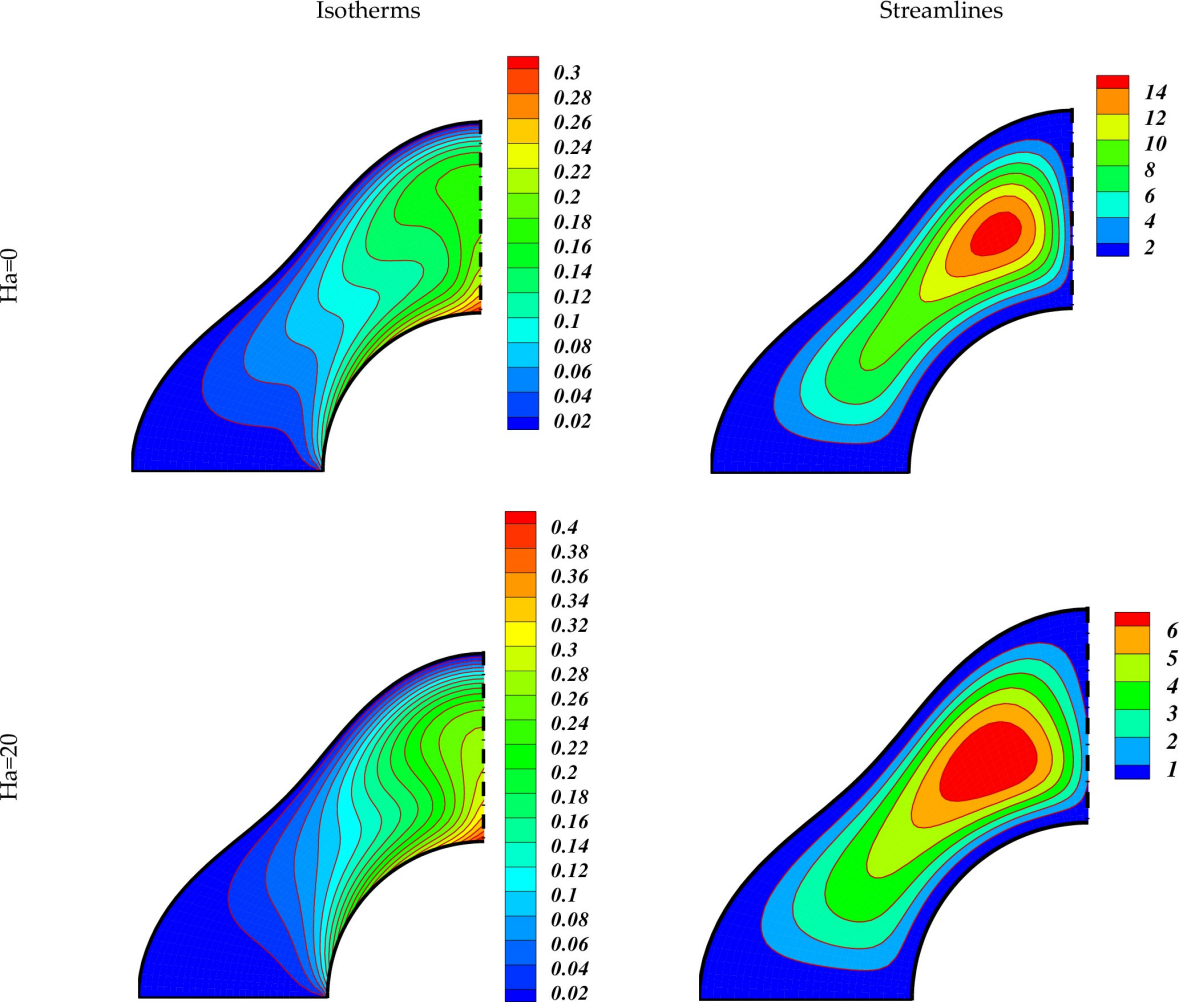
Impact of Ha on the flow when *Ra* = *10*^*5*^,*Rd* = *0*.*8*,*Da* = *100*.

We currently deliberate the outcomes of the current exploration from the significant sketched graphical features. Here, the various important characteristics of MHD non-Darcy hybrid nanofluid over a porous cylinder are investigated and discussed through contours and 3-D plots with changing the strength of buoyancy forces (varying Ra), magnetic field (varying Ha), medium porosity (varying Da), and radiation parameters (varying Rd). The impact of varying Da on the hybrid nanofluid flow is exhibited in Fig 3. This figure has two plots. The left one describes the hybrid nanofluid flow for Ha = 0, while the right plot shows the flow for Ha = 20. The other parameters values used are Ra = 10^3^, Rd = 0.8. The dark red dotted lines represent the flow for Da = 0.01 while the blue solid lines represent the flow at Da = 100 in both these plots. The larger Da means the higher medium porosity, which allows the hybrid nanofluid to flow easily. We see that when Ha = 0, the hybrid nanofluid flow is very stronger in the middle region, and becomes weaker as we move to the outer region. By increasing the value of Ha to 20, the flow becomes weaker due to the greater Lorentz force which delays the hybrid nanofluid flow. Fig 4 displays the effect of varying Lorentz forces on the Isotherms and Streamlines of the hybrid nanofluid through contour plots. The first two plots of Fig 4 are designed for Ha = 0 whereas the last two plots are portrayed for Ha = 20. The estimations of the further parameters used are Ra = 10^5^, Da = 0.01, Rd = 0.8. It is found that the strength of the Isotherm contours augments with the growing magnetic effect. Thus the heat of the inner wall enhances due to the inter-particle collisions with higher magnetic fields. The right plots show that the strength of the vortexes of the Streamlines decreases with augmenting Ha. Hence the convective hybrid nanoliquid flow reduces with the higher Lorentz forces. Fig 5 depicts the impact of varying Lorentz forces on the Isotherms and Streamlines of the hybrid nanofluid through contour plots. In this case, the permeability of the porous cylinder has increased by taking the value of Darcy number, Da = 100. The first two plots of Fig 5 are designed for Ha = 0 whereas the last two plots are represented for Ha = 20. The estimations of the other parameters used are Ra = 10^5^, Rd = 0.8. It is found that the strength of the Isotherm contours enhances with the augmenting magnetic field which shows that the heat of the inner partition enhances due to the inter-particle collisions with higher magnetic fields. The comparison with the contour plots of the Isotherms in Fig 4 shows that the enhancement in the temperature of the internal wall enhances with the rising Ha by a small amount in this case. Thus it can be said that at higher Da, the rate of growth of the hybrid nanofluid temperature is small with the expanding Ha. The right plots show that the strength of the vortexes of the Streamlines decreases with rising Ha. Hence the conductive hybrid nanoliquid flow increases with the higher Lorentz forces. The comparison of the contour plots of the Isotherms displays that the convective hybrid nanoliquid flow enhances by a greater amount with the increasing Da at Ha = 0.

The following analytical expression for Nu_ave_ is obtained:

$$\begin{array}{l} Nu_{ave}=4.97+2.59Rd+2.96\log(Ra)+0.18Da^{*}-0.29Ha^{*}\\ +0.06RdDa^{*}-0.15RdHa^{*}+0.2\log(Ra)Da^{*}-0.39\log(Ra)Ha^{*}-0.2Da^{*}Ha^{*} \end{array}$$ ((15))

This expression shows that Nu_ave_ is the function of Rd, Ra, Da and Ha.

**Fig 6:**
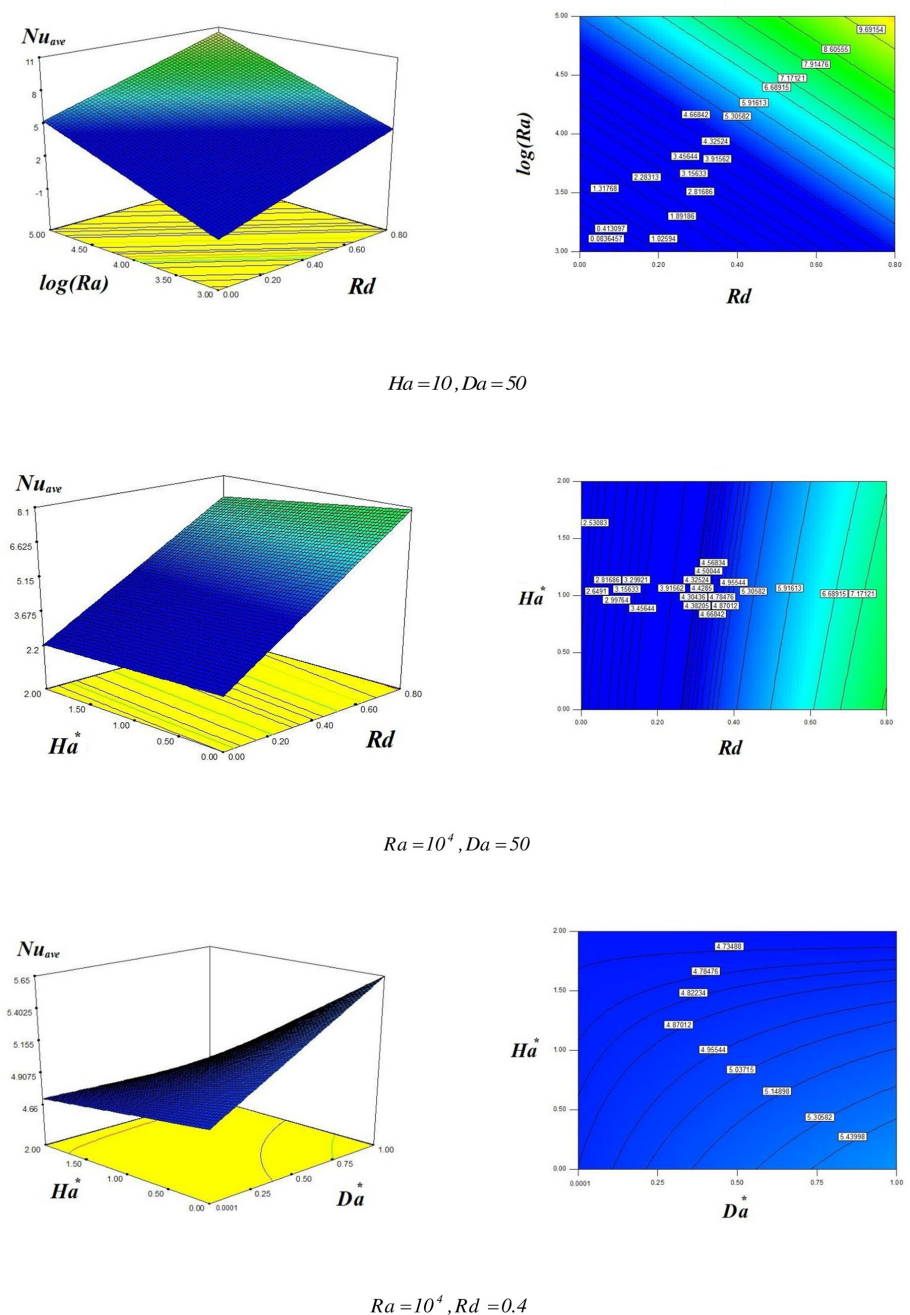
Influences of *Ra*,*Ha*,*Da*,*Rd* on *Nu*_*ave*_.

The influence of the changing estimations of Ha, Da, log (Ra) and Rd on the average Nusselet number Nu_ave_ is displayed in Fig 6. There are six plots in Fig 6. The first two plots show the difference of Nu_ave_ with the rising estimations of log (Ra) and Rd. The estimations of the other parameters used are Ha = 10 and Da = 50. From these plots, it is observed that Nu_ave_ enhances with a uniform rate with the augmenting values of both log (Ra) and Rd. Thus the higher buoyancy forces and increasing strength of radiation source amplifies the convective heat transfer flow. The middle two plots depict Nu_ave_ as a function of Ha and Rd. The other parameters are taken as Ra = 10^4^ and Da = 50. It is evident from these plots, that Nu_ave_ enhances with rising radiation parameter while drops with the increasing Hartmann number. The rate of escalation of Nu_ave_ with higher Rd is much bigger as related to the rate of decrease with augmenting Ha. Thus the convective heat energy transmission decreases with the higher Lorentz forces which constrict the hybrid nanofluid flow. The last two plots depict Nu_ave_ as a function of Ha and Da. The estimations of the further parameters used for these plots are Ra = 10^4^ and Rd = 0.4. These plots exhibit that Nu_ave_ remains almost constant with the expanding Ha, whereas augments with the increasing Da. Thus the increasing porous medium permeability allows the hybrid nanofluid to flow easily and hence enhances the convective energy transfer.

## 6. Conclusions

This section is devoted to concluding the current research work. The hydro-magnetic behaviors of the hybrid nanofuid flow are investigated with the varying strength of Lorentz force and porousness of the permeable medium through contour plots. The variation of Nusselt number Nu_ave_ with the increasing strength of Renold number Ra, Darcy number Da, radiation parameter Rd, and magnetic parameter Ha. is analyzed through 3-D plots. The main findings of this research work are:

- The temperature of the hotter wall rises with the greater Hartmann number due to the increasing inter-particle collision of the hybrid nanofluid. This enhancement in temperature with rising Ha is more dominant at smaller Darcy number Da.
- The augmenting Lorentz forces due to the higher estimations of Hartmann retard the hybrid nanoliquid flow and hence enhance the conduction.
- The increasing Da causes to enhance the strength of the Streamlines more effectively in the absence of Lorentz forces.
- The agreement between the current and previously published results validates the numerical simulation performed through CVFEM.
- An analytical expression obtained for Nusselt number Nu_ave_ shows its functional dependence on Ra, Darcy number Da, radiation parameter Rd, and magnetic parameter Ha.
- The average Nusselet number (Nu_ave_) enhances with the expanding Ra, Da and Rd, while decreases with the increasing Ha.
